# Use of contrast media in diagnostic imaging: medico-legal considerations

**DOI:** 10.1007/s11547-015-0549-6

**Published:** 2015-06-17

**Authors:** C. Pomara, N. Pascale, F. Maglietta, M. Neri, I. Riezzo, E. Turillazzi

**Affiliations:** Section of Legal Medicine, Department of Clinical and Experimental Medicine, University of Foggia, Ospedale Colonnello D’Avanzo, Via degli Aviatori, 1, 71100 Foggia, Italy

**Keywords:** Contrast media, Adverse reaction, Informed consent, Off-label use, Medical liability

## Abstract

Contrast media (CM) are used in imaging techniques to enhance the differences between body tissues on images. The ideal contrast medium should achieve very high concentration in the tissues without producing any adverse effects. Unfortunately, this has not been possible so far and all CM have adverse effects. The increasing use of CM is likely to give rise to a wide range of pitfalls, including compliance with and appropriateness of indications for the use of CM themselves, the choice of the ‘best’ contrast agent, off-label use, evaluation of special populations of patients, and competence to tackle emergency scenarios following the administration of CM. Even more prominent, and potentially more important, is the issue of informed consent which brings with it a duty to inform patients awaiting the administration of CM with regard to the nature of the procedure, the existence of alternative procedures, the extent of the risks relating to the use of CM and, finally, the risks relating to refusal of the procedure. All these issues may give rise to concerns about liability for failure to offer adequate information to patients or to carefully evaluate and balance the potential risks and benefits of the procedure or, finally, for being unprepared in the event of adverse reactions to CM, especially when these are severe and life-threatening. Educational and training programmes for radiologists are likely to shape change in the medical liability environment in the coming years.

## Introduction

Contrast media (CM) enhance the quality of images, revolutionizing the radiologist’s ability to differentiate soft-tissue densities. Ideally, CM should achieve very high concentration in the tissues without producing any adverse effects. Unfortunately this has not been possible so far and all CM have adverse effects [1].

In the last decades procedures employing CM have rapidly increased. Significant improvements in the composition of CM during the past few decades have made them safer and better tolerated. Nonetheless, risks associated with CM have not been eliminated: varying degrees of adverse reactions continue to occur, and, in some situations, their use is problematic [2–7]. The reported rates of severe reactions to CM are quite low; however they can quickly became life-threatening and lead to death [8–11]. Radiologists have been challenged in many ways by the issues arising from the increasingly widespread use of CM. The aim of this review is to define those medico-legal issues we believe to be most pertinent to the use of CM in diagnostic imaging as we know it today.

## Informed consent

The basic concept of informed consent is familiar to radiologists [12, 13]; radiologists are aware that no diagnostic investigation can be performed without the patient’s valid consent and that the patient must be given adequate information and sufficient data to be able to make an informed decision about the examination. However, failure to obtain proper informed consent is a frequent cause of lawsuits. A recent United States nationwide research showed that the commonest causes of medical malpractice suits against radiologists were diagnostic errors and procedural complications, followed by inadequate communication with either patient or referring physician [14]. Data coming from European countries confirmed that lack or invalidity of informed consent is highly problematic in radiological practice [15–17]. Interestingly, O’Dwyer et al. [17] reported that more than 50 % of the radiologists interviewed were unhappy with their policies for obtaining informed consent.

Effective physician–patient communication is an enduring process. Consent to medical intervention infers a profound relationship between patient and physician which cannot be compressed into a short encounter. It is a process which implies the doctor’s duty to inform patients of the benefits and potential risks of the treatment options, to answer their questions openly and honestly, to help patients in their choice and, finally, to accept that choice [18].

Special scenarios and caveats regarding the practice of radiology and particularly the use of CM need to be discussed.

One key question is that the patient-radiologist relationship tends to be brief and episodic, so that radiologists are unlikely to have an established relationship with the patient [19], and may not feel comfortable discussing the risks and complications of their procedures with the patient [20]. Undoubtedly, time is a critical issue for radiologists in the informed consent process [21, 22]. In their survey on current European practice, O’Dwyer et al. [17] report that, despite the fact the appropriate time for obtaining informed consent is considered to be more than 24 h prior to the procedure, very often it is obtained on the same morning or immediately before the procedure itself. However, patients have to be provided with adequate time to consider the information they have received. Finally, also the time that radiologists spend with patients is critical in ensuring that patients are satisfactorily informed [23].

Another key point is the amount of information, especially with regard to risks, that should be given to patients; and in radiological practice the use of CM exacerbates the issue [24–26]. The appropriateness of the use of CM, any alternative procedure, the risks and benefits of not undergoing the proposed diagnostic or interventional procedure with CM have to be outlined in the informative process. On exploring patients’ attitude towards informed consent for CM, Hopper et al. [27] pointed out that most people desire to be informed before a contrast injection. It is generally argued that excessive and detailed information about the risks of the procedure may increase the levels of anxiety in a patient awaiting administration of CM. On the contrary, balanced communication about the risks seems to reduce anxiety levels [28–30]. In the ACR manual on CM [13] it is stated that ‘because of the documented low incidence of adverse events, intravenous injection of contrast media may be exempted from the need for informed consent, but this decision should be based on state law, institutional policy, and departmental policy’. Other authors seem to take the same view [31].

Contrarily, we believe that the low statistical frequency of complications, specifically those which are life-threatening, does not exempt radiologists from the duty to inform patients of these specific risks. Obtaining consent for all radiological CM procedures is an ethical duty, regardless of the nature of the agent used and the incidence and severity of the possible adverse events. A correct informative process is one in which physicians communicate the gist of the message to the patients, i.e., that there is some risk involved in the use of CM. Communicating the gist without referring to statistics, tailoring information to the patient’s needs and facilitating people’s understanding are essential elements of informed consent when radiologists are using contrast agents.

Finally, the form of informed consent (written or verbal) that is mainly based on state laws, and institutional and departmental policies. Some general considerations on the form of communication can be of interest. Efforts to improve information delivery and patient knowledge are widely reported in the literature as an important prerequisite for informed consent. There is wide agreement that a multimedia approach combining videos, verbal communication, audio tape, pamphlets, and interactive methods of communication may improve the patient’s comprehension and participation in the decision-making process [32]. The readability of informed consent forms is a critical issue [33] as many informative leaflets are written in such a way as to be incomprehensible to the average patient.

The above-mentioned criticisms arise particularly in emergencies where an inherent difficulty with obtaining adequate informed consent exists [34, 35].

## Off-label use

The first point concerns the definition of off-label contrast medium (OLCM) which depends on the regulatory environment of different countries. In general terms, off-label use means use other than the originally tested and licenced indications, and the obtaining of licences depends on drug laws which may vary from country to country.

In the European normative framework, CM fall within the definition of medicinal product (EC Directive 2004/27/EC 31 March 2004): ‘Any substance or combination of substances which may be used in or administered to human beings either with a view to restoring, correcting or modifying physiological functions by exerting a pharmacological, immunological or metabolic action, or to making a medical diagnosis’ [36, 37]. In the United States off-label use generally means that a medical product is not administered for the specific use approved by the food and drug administration (FDA) and listed in the drug-labelling information. To sum up, off-label use of a medical product is its prescription and use in a manner and for purposes other than those approved by competent authoritative agencies and national drug laws. Since these agencies regulate only the labelling of the medical products and do not regulate the practice of medicine, the off-label use of drugs and medical products is becoming increasingly common [38].

To fully understand the medico-legal implications of OLCM, the differences existing between CM and other drugs have to be taken into account. As outlined by Thomsen in his editorial, CM “are not designed to have therapeutic effects and they are administered in one dose, under medical supervision, and provide their effect under the principles of physics, not those of pharmacology” [39]. According to current regulations CM are officially approved for some specific uses and for body areas; their use in imaging the rest of the body is considered “off-label” [39]. For all these reasons, often the approved indications do not match the real clinical and diagnostic needs and in daily practice the off-label use of the CM is extremely common, mostly involving gadolinium-based contrast agents (GBCAs), especially in MR angiography, cardiac and paediatric applications [36, 40].

The most striking example is contrast-enhanced ultrasound (CEUS), particularly in the paediatric population, that is increasingly practised since it may reduce the use of ionizing radiations, and nowadays it is an established technique for many organs. In Europe, the use of CEUS is approved for a limited number of indications in adults [41]. Cardiac application of ultrasound contrast agent is approved in the United States, but it is not known when or if ultrasound contrast agents will be approved for non-cardiac applications there [42]. No-labelled use is approved in the paediatric population [43]. There is widespread use in Europe [44], and there are ongoing efforts by the Society for Paediatric Radiology and International Contrast Ultrasound Society to push for paediatric CEUS in the United States [45]. Therefore it may be expected that CEUS will be increasingly used throughout childhood [46].

Liability profiles may result from OLCM use since, despite widespread clinical practice, it is still formally outside the regulatory boundaries [37]. This incurs greater risks for the radiologist. It is known, for example, that with increasing off-label use, significant differences have not been observed in the incidence of severe adverse reactions between approved and unapproved use of CM [39]. Nevertheless, in the case of adverse reaction, especially if severe and life-threatening, the radiologist must demonstrate that the off-label use of that CM was fully supported by scientifically valid evidence and that there were no contraindications for the safety of the patient. This is a general requirement in off-label use of medical products which sharpens the need for strong scientific support for this practice. Making a clear distinction between CM off-label uses that are well supported by the best scientific evidence available and those that are not is the Gordian knot of the issue [47]. This concept is strongly supported by the statements of many scientific societies and regulatory bureaus. The FDA states that if a physician uses an off-label drug or medical device, he or she should base judgment on sound medical evidence and should maintain a record of the products used and their effects [48]. In a similar vein is the position of the Society of Interventional Radiology which supports the lawful use by a physician of a drug product for an unlabelled indication when such use is based on sound scientific evidence and/or sound medical opinion [49]. The second vital point, again with regard to the professional responsibility of the radiologist and possible requests for damages arising from the off-label use of the CM, is the information supplied to the patient prior to doing the exam. Since the radiologist uses a CM which the regulatory body has not stated is safe and effective for that specific use, we believe that he/she is obliged to provide exhaustive information to patients and obtain formal consent before using an OLCM. Conflicting conceptual positions arise around the thorny issue of informed consent in the off-label use of medical products. Some authors agree that it is not acceptable for a physician to neglect to tell patients of a medical product’s off-label status while some others have contended that off-label status is irrelevant to the actual medical risks posed [50–52]. While aware of the specific nature of the CM with regard to traditional medicines, we consider that the radiologist is still obliged to inform the patient of the off-label use of the CM, to explain the clinical and scientific reasons supporting it, to highlight the advantages in terms of effective diagnosis, to explain any alternative diagnoses and finally to obtain informed consent to the off-label use of the CM [40].

In conclusion, some key points can be drawn. If radiologists use a contrast agent for non-approved purposes, they are responsible for balancing the benefits and the potential negative effects of such use. They must also base its use on firm scientific rationale and on sound medical evidence when alternative labelled products are not of equally proven efficacy, and fully inform patients of the potential adverse effects of the product.

Finally, it should also be pointed out that it is the duty of radiologists to report any suspect adverse reaction associated with OLCM use to local/national authorities responsible for drug safety monitoring. Radiologists should be made aware that the reason for collecting information on OLCM use is to ensure the highest standard of patient safety, possibly further to a review by ‘ad hoc’ local and national committees of incidents involving OLCM use and to subsequent appropriate actions.

## Medical liability

The principles regulating medical liability differ from country to country and between common law and civil law systems, and malpractice in radiology varies across the globe [53]. Overall, the percentage of medical malpractice lawsuits involving radiologists has been estimated to range from 5 to 12 % [54, 55]. Diagnosis seems to be the major pitfall for radiologists [14]. In daily practice, around 3–5 % of radiological analyses contain errors [56–60]; errors in interventional procedures and adverse events occurring during a radiological examination take second place in this negative ranking [61].

The issue of medical liability and malpractice is not unique to radiologists; however, some key points have to be stressed, focusing particularly on the use of CM [2, 3, 62, 63].

### Indication for the examination, and specifically the use of contrast media

Except for screening tests, radiologists generally receive the requests for examination from a referring physician. The decision of whether or not to conduct the examination and whether CM should be used is left to the radiologist [63]. Radiologists may fail to adequately check the appropriateness of the indication for the use of CM and to weigh up the diagnostic/therapeutic benefits and the possible risks. Existing guidelines may strongly assist the radiologist in making the most appropriate imaging decision for a specific patient [64, 65].

### Investigation into medical history

Careful investigation into the patient’s medical history with special attention to eventual previous reaction to CM is critical. The radiologist who decides to use a contrast agent should investigate whether the patient has had a history of allergy and elicit from the patient any information regarding allergies through specific questions rather than simply relying on the patient, for the latter could overlook clinical significant details (i.e., previous life-threatening reaction, episodes of bronchospasm or asthma, hypotension or shock from an underlying illness, history of allergies, and other medical conditions, including possible renal impairment, heart failure, diabetes, hyperthyroidism, drug addiction, etc.) [65]. It is noteworthy that risk is increased sixfold by a history of adverse reaction, six to tenfold by asthma, and to a considerable extent by a history of allergic reactions to other drugs [66, 67].

### Careful attention to special populations

Even more attention is required before administering CM to special populations.

#### Pregnant and breastfeeding women

Beyond the risks related to the exposure of the foetus to ionizing radiations and high magnetic fields, the administration of CM may be a further hazard for the foetuses and neonates [68].

CM cross the human placenta, thus entering the foetus. They can also be secreted into milk during lactation. Mutagenic effects have not been described after administration of gadolinium or iodinated CM [68–70]. However, the lack of adequate and well-controlled studies regarding these possible effects in humans has induced the ACR to recommend the use of iodinate-contrast media in pregnant women only when: ‘(1) the information requested cannot be acquired without contrast administration. (2) The information needed affects the care of the patient and foetus during the pregnancy. (3) The referring physician is of the opinion that it is not prudent to wait to obtain this information until after the patient is no longer pregnant’ [64]. The same caution is required when deciding to use GBCAs. Also, the updated version of ESUR guidelines [65] recommends that the use of iodine-based CM in pregnant women should be limited only to exceptional circumstances and that following their administration, thyroid function should be checked in the neonate during the first week. Regarding the use of gadolinium-based contrast agents, the revised guidelines recommend the administration of the smallest possible dose of the most stable GBCAs only when there is a very strong indication.

Both iodinate- and gadolinium-based CM are thought to be safe for mothers and children. However, full information about the mother with regard to the possibility of temporarily stopping lactation is suggested [13]. ESUR guidelines state that ‘breast feeding may be continued normally when iodine-based agents are given to the mother. Breast feeding should be avoided for 24 h after contrast medium if high risk agents are used’ [65]. A recent position paper of the Italian Society of Radiology, the Italian Society of Paediatrics, the Italian Society of Neonatology and the task force on breastfeeding, Italian Ministry of Health [71] states that ‘breastfeeding is safe for the nursing infant of any postconceptional age after administration to the mother of all iodine-based contrast media and most gadolinium-based contrast media. As a precaution, gadolinium-based agents considered at high risk of nephrogenic systemic fibrosis should be avoided in the breastfeeding woman. There is no need to discontinue breastfeeding for 12–48 h after the administration of contrast media and no use in expressing and discarding breast milk following the imaging’.

In conclusion, although the regulatory statements and clinical practice for the use of contrast media in pregnancy and lactation differ [70], caution is strongly advised and radiologists must be aware that the use of contrast media in pregnant women must be limited to cases when the benefits outweigh the potential risks. Accurate information and written informed consent to the procedure are strongly advised.

#### Patients who take metformin

They need special attention before the administration of CM since their use, and specifically iodine-based CM, may increase the risk of lactic acidosis patients taking metformin with known, borderline, or incipient renal dysfunction [72]. Management varies according to the recurrence and/or severity of renal impairment: no discontinuation of metformin nor creatinine control in patients with normal renal function and no comorbidities is required; discontinuation at the time of examination and for 48 h in patients with multiple comorbidities and apparently normal renal function, followed by the reassessment of renal function before restarting metformin is required. In patients with renal dysfunction, metformin should be suspended at the time of the contrast injection, and cautious follow-up of renal function should be performed until safe reinstitution of metformin can be assured. It is not necessary to discontinue metformin prior to gadolinium-enhanced MR when the amount of gadolinium administered is in the usual dose range [64]. Taking the same view, ESUR guidelines advocate increased attention depending on the existence and severity of renal failure for iodinate CM. No special precautions are requested for GBCAs [65].

#### Patients with previous renal insufficiency

They are at greater risk of developing contrast-induced nephropathy (CIN) than patients whose function is normal. CIN is a well-described iatrogenic effect of the use of iodinate-contrast medium that occurs more frequently in patients with previous renal insufficiency [73]. These patients deserve special attention in the decision-making process leading to the use of CM. Radiologists must be fully informed and must use evidence-based protocols [74]. Statements from scientific associations provide specific recommendations on how to manage these patients [64, 65].

### Premedication

Premedication is critical in possible liability scenarios linked to the use of CM. In some circumstances premedication is required. We refer to patients with previous reactions to CM for whom a pre-treatment regimen, including administration of corticosteroids with or without antihistamines or other medications, is thought to be safe. However, acute adverse reactions (breakthrough adverse reactions) are known to occur even when the patient is pre-medicated [75–77]. A recent review of the existing literature shows that in unselected patients, the usefulness of premedication is doubtful, and data supporting the use of premedication in patients with a history of allergic reactions are lacking [78]. Local institutional policies may exclude the use of CM in patients with prior moderate or severe reactions and in pre-medicated patients whose previous reactions were mild [77]; however, the Contrast Media Safety Committee of ESUR considers the use of premedication, although evidence of its effectiveness is limited in patients with previous moderate or severe acute reactions [65].

Several different premedication regimens have been proposed to reduce the frequency and/or severity of reactions to contrast media. The possible switch to other contrast agents, and the strict observation of the patients (20/30 min after the contrast medium injection, having drugs and equipment for resuscitation quickly and readily available) are also suggested as precautions for radiologists [79]. However, radiologists are reminded that they should be prepared to deal with the possible reactions [64, 65].

In conclusion, the take-home message is that radiologists must be wary of reactions in patients with a previous history of life-threatening reaction, bronchospasm or asthma, hypotension or shock from an underlying illness and allergies and be aware that premedication does not completely eliminate the risks of severe and life-threatening reactions in those patients. Staying alert and adequately prepared to administer resuscitation and supportive treatments are essential and cannot be replaced by premedication [76, 78]. The statement that ‘physicians who are dealing with these patients should not rely on the efficacy of premedication’ [78] must be kept in mind by radiologists when deciding whether or not to use CM in patients with prior reaction.

### Appropriateness and timeliness of adverse reaction management

Finally, radiologists can be sued if the management of the adverse reaction induced by a contrast agent is not prompt and correct. Rapid identification of alarming, life-threatening symptoms and adequate therapeutic strategies are recommended according to the kind and the severity of the adverse reaction [80, 81]. However, knowledge of the management of acute contrast reactions is lacking among radiologists [82] and training and educational programmes are of paramount importance to help them to improve their performance in emergency scenarios following the administration of CM [82, 83].

## Conclusions

The increasing use of CM is likely to give rise to a wide range of pitfalls, from compliance with and appropriateness of indications, to the choice of the ‘best’ contrast agent. Moreover, off-label use, evaluation of special populations of patients, and readiness to deal with emergency scenarios following the administration of CM are some of the most challenging issues for radiologists. Even more prominent, and potentially more important, is the issue of informed consent which implies a duty to inform patients awaiting the administration of CM of the nature of the procedure, the existence of alternative procedures, the extent of the risks related to their use and, finally, the risks of refusing the procedure.

All the above-mentioned issues may give rise to concerns about liability for failure to offer adequate information to patients or to carefully evaluate and balance the potential risks and benefits of the procedure or, finally, for being unprepared in the event of adverse reactions to CM, especially when severe and life-threatening. Educational and training programmes for radiologists are likely to shape change in the medical liability environment in the years to come.
